# Hybrid Telehealth Pediatric Constraint Induced Movement Therapy Compared to In-person Intervention: A Case Series

**DOI:** 10.5195/ijt.2023.6567

**Published:** 2023-12-12

**Authors:** Teressa Garcia Reidy, Nicole Whiston Andrejow, Erin Naber, Joan Carney

**Affiliations:** 1 Specialized Transition Program, Kennedy Krieger Institute, Baltimore, MD, USA; 2 Physical Medicine and Rehabilitation, Johns Hopkins University School of Medicine, Baltimore, MD, USA

**Keywords:** Constraint Induced Movement Therapy, Hemiplegia, Hybrid intervention protocol, Pediatrics

## Abstract

Constraint induced movement therapy is an established, evidence-based intervention for children with hemiplegia. This case series describes the feasibility and clinical opportunities of using a hybrid telehealth and onsite model to deliver pediatric constraint induced movement therapy during the COVID-19 pandemic. These cases support that a hybrid model had a similar effect on upper extremity improvement compared to a traditional, in-person model and may be an option when access to in-person care is not available.

Research supports the clinical use of in person constraint induced movement therapy (CIMT) for children and infants with hemiplegic cerebral palsy to improve functional use of the affected upper extremity and bimanual skills (Baker et al., 2022; Novak et al., 2020; Ramey et al., 2021; Reidy et al., 2012; Reidy et al., 2017; Walker et al., 2022). Historically, the large onsite time commitment and limited access to specialty programs has been a barrier to accessing adult and pediatric CIMT (Pickett et al., 2007). In pediatric CIMT, parent hesitation and concerns for lost work and lost wages compounds this limited access (Pietruszewski et al., 2020).

In the context of the COVID-19 pandemic public health emergency, changes in reimbursement policies and licensure restrictions allowed for the rapid expansion of the use of telerehabilitation (Tanner et al., 2020). The efficiency of this mode of service delivery allows for improved access to specialized care by minimizing barriers of transportation and distance, while optimizing valuable clinician time. Emerging evidence supports that telerehabilitation can be an effective treatment option for children with cerebral palsy to address their interdisciplinary goals (Onal et al., 2021; Tamboosi et al., 2021; Tanner et al., 2020).

While telehealth utilization has recently increased, there is currently limited evidence describing these programs in pediatric rehabilitation (Onal et al., 2021; Pietruszewski et al., 2020; Shierk et al., 2021). A systematic review by Knapp et al. (2023) identified a need for research in telehealth delivery models and efficacy of various models to ensure evidence-based clinical practice. This review reported that available evidence was limited to mostly qualitative research to date (Knapp et al., 2023). Despite the small body of evidence, studies exploring the feasibility of telehealth home programs and effectiveness of parent coaching models in rehabilitation have demonstrated positive patient and caregiver feedback (Beckers et al., 2020; Verhaegh et al., 2022). In addition, qualitative physical therapy research has reported benefits to hybrid models of care (a protocol with a combination of onsite and telehealth visits) in pediatrics that include improved patient communication, better understanding of the home environment and improved access to and continuity of care (Hall et al., 2022).

To date there have been limited pediatric studies exploring telehealth models of CIMT (Shierk et al., 2021) but an adult hybrid model has demonstrated increased functional ability and greater attendance in adults in the chronic phase of stroke recovery (Smith & Tomita, 2020).

There is a call to research topics such as feasibility, methods of engaging families in therapeutic activities and exploring different platforms for telehealth service delivery (Onal et al., 2021; Ownsworth et al., 2018). In addition, developing clinically reproducible treatment protocols is essential to compare outcomes of telehealth interventions to their in-person counterparts. Given the clinical and lab research that has been published about CIMT and that it has demonstrated success delivered via telehealth in an adult trial, it was a logical model to attempt with pediatrics in the telehealth sphere.

This paper aims to describe the feasibility and clinical experiences of using hybrid telehealth to deliver pediatric constraint induced movement therapy during the COVID-19 pandemic. In addition, this case series will present the outcomes of hybrid telehealth CIMT and subsequent in person CIMT models in two pediatric clients.

## Methods

Three existing protocols for CIMT existed in the clinic for several years prior to the COVID-19 pandemic. The Traditional CIMT Protocol (Reidy et al., 2012) involved use of a long arm cast worn for 24 hours a day for 16 days while the patient received 3 hours of combined occupational (OT) and physical therapy (PT) daily. The cast was then removed, and intensive bimanual therapy was the focus for 3 hours daily for the remaining 5 days. In the Modified CIMT Protocol (Whiston et al., 2019) patients enrolled in a 4-week interdisciplinary day hospital program were casted and received CIMT intervention during daily 1-hour OT sessions. Patients in this protocol received up to 6 hours of daily interdisciplinary intervention. The Infant Protocol (Reidy et al., 2017) used a thermoplastic or soft splint worn for a total of 2 hours daily for 20 sessions. Intervention was done on-site, in person, with the constraint by the therapist for 1 hour per day. Caregivers completed an additional hour of intervention with the constraint donned at home with a customized home program. Bimanual activities were encouraged when the cast was doffed. In all protocols, onsite sessions were comprised of fine motor, play and ADL activities. A coaching model (Hanft, et., 2004) was utilized to train families. Coaching interventions are dynamic interactions between the practitioner and the caregiver in which the practitioner assesses the caregivers' understanding of how to facilitate improved function, provides feedback and then reflects upon performance with the caregiver (Rush & Shelden 2011). The therapist and caregiver develop a plan for the session to address specific actions and then implement that plan with ample opportunity for the therapist to observe and facilitate refinement of caregiver's skills (Rush & Shelden, 2011). Reflection and feedback is a defining attribute of coaching that makes it unique and distinctive from training or consultation (Rush & Shelden, 2011). Reflection provides a context for the practitioner to ask the caregiver what was effective or not during the action phase. They can then collaborate to brainstorm alternatives. This further improves the skill set and knowledge of the caregiver to facilitate improved independence within the child's functional capacity (Rush & Shelden, 2011).

These two cases initially completed a hybrid telehealth Infant CIMT Model due to the pandemic emergency. Both returned for a subsequent in-person intervention. One returned for an in-person Traditional CIMT Model and the other patient returned for a Modified CIMT Program due to the patient's ambulation status.

Within the hybrid model, the patients received a combination of telehealth and onsite intervention. During telehealth sessions, the therapist led the caregivers in directing the intervention including selecting the toys and positioning, grading therapeutic activities, providing and modeling positive reinforcement and cuing to maintain engagement. During onsite intervention the therapist led the intervention and caregivers returned demonstration and practiced skills. Cast fabrication and outcome measures were performed on-site for both patients. Therapists were not blinded to treatment as this was routine clinical care. The Quality of Upper Extremity Skills Test (QUEST) (DeMatteo et al., 1993) and the Assisting Hand Assessment (AHA) (Krumlinde-Sundholm et al., 2007) or the Mini-Assisting Hand Assessment (Mini-AHA) (Greaves et al., 2013) per patient age were the standardized assessments used to measure changes in upper extremity and bimanual function. QUEST scores can be interpreted with caution as it is not normed for children under 18 months. Although the QUEST was not standardized for this age group the therapists found the Dissociated Movements and Grasps subtests clinically useful. Since it is an observational tool that looks at specific and coordinated movements of the upper extremity it provided a baseline and post intervention numerical score to quantify change. In this age group there is a lack of standardized measures that can provide reliable numerical documentation of these skills.

## Case Descriptions

Patient 1 was a female infant with hemiplegic cerebral palsy secondary to in utero intraventricular hemorrhage (right side and basal ganglia involvement) with a ventriculo-peritoneal shunt. She was 8 months old at the time of her first admission and completed the clinic's established Infant CIMT Protocol (Reidy et al., 2017) in a hybrid model. She completed her evaluations and one of 18 treatment days onsite and 17 of 18 treatment days via telehealth. She was 24 months old at the time of her second admission and completed the clinic's established Modified CIMT Protocol (Whiston et al., 2019) in person because she was not yet walking steadily. She completed her evaluations and 18 of 18 treatment days onsite.

Patient 2 was a male infant with left hemiplegic cerebral palsy and polymicrogyria. He was 8 months old at the time of his first admission and completed the Infant CIMT Protocol (Reidy et al., 2017) in a hybrid model. He completed his evaluations and nine of 18 treatment days onsite and nine of 18 treatment days via telehealth. He was 24 months old at the time of his second admission and completed the clinic's Traditional CIMT protocol (Reidy et al., 2012) in person. He completed his evaluations and 22 of 22 treatment days onsite.

## Results

### Patient 1

**Table:** 

	*Admission 1: Hybrid*				*Admission 2: Onsite*		
*Assessment*	*Subtest*	*Initial*	*Discharge*	*Assessment*	*Subtest*	*Initial*	*Discharge*
*Mini-AHA* ^*^	n/a	9	20	*AHA* ^*^	n/a	45	55
*QUEST* ^	Dissociated Movements	45.32	57.82	*QUEST* ^	Dissociated Movements	69.36	76.56
	Grasps	26.32	31.58		Grasps	40.74	59.26
	Weight Bearing	NT	NT		Weight Bearing	NT	NT
	Protective Extension	NT	NT		Protective Extension	NT	NT

*Note*.

* Logit scores are reported;

^ Standardized scores are reported

### Patient 2

**Table:** 

	*Admission 1: Hybrid*				*Admission 2: Onsite*		
*Assessment*	*Subtest*	*Initial*	*Discharge*	*Assessment*	*Subtest*	*Initial*	*Discharge*
*Mini-AHA* ^*^	n/a	33	46	*AHA* ^*^	n/a	46	55
*QUEST* ^	Dissociated Movements	40.62	59.38	*QUEST* ^	Dissociated Movements	56.26	73.44
	Grasps	31.58	47.36		Grasps	44.44	62.96
	Weight Bearing	NT	NT		Weight Bearing	57.9	76
	Protective Extension	NT	NT		Protective Extension	75	83.34

*Note*.

* Logit scores are reported;

^ Standardized scores are reported

## Discussion

Formal statistical analysis was not performed for the above results due to the small sample size and variations in assessments, however several observations were made when reviewing the assessment results of these cases.

The subtest scores for the QUEST are reported due to variability in total score dependent on the inclusion or exclusion of subtests based on patient age and treatment protocol. Both patients improved scores on all subtests after intervention regardless of delivery method. For the first patient there was a steady increase in scores for the Dissociated Movement and Grasp subtests of the QUEST. The patient did not demonstrate a reversion of skills to baseline when tested at the beginning of the second admission. It appears that the second intervention episode built on the skills retained from the first admission. While the second patient had a slight decrease in scores in Dissociated Movement and Grasp subtests between admissions, the gains during the second admission reflected overall improvements in arm function with repeated CIMT.

Both patients were tested with the Mini-AHA and then AHA due to their ages and testing criteria. Currently, there is no way to directly compare the scores of these assessments. There is also no validated way to convert the score of one to the other as the child ages. The data is presented so the reader can see the placement on the logit scale for each admission for some comparability. A clinically relevant change of at least 5 points on the logit scale (Krumlinde-Sundholm, 2012) was achieved for both patients for each admission.

The clinical gains of these patients mirror results these authors have observed with over 15 years of experience using in-person CIMT protocols. When comparing these data to historical onsite clinical data, these patients had parallel achievements and similar outcomes. These cases support that a hybrid model had a similar effect of upper extremity skills achieved compared to a traditional, in person model as reflected by the AHA score changes. It may be a feasible option when access to in-person care is not available. In addition, hand skill assessments that span a large age range that can document progress over time would be beneficial for children with hemiplegia and are scarce in pediatric rehabilitation research (Smegal et al., 2023). Although correlation of the mini-AHA and the AHA have not yet been validated they are similar measures that look at similar bimanual skills (L. Krumlinde-Sundholm, personal communication, February 23, 2023).

Although this intervention was trialed with a small sample the authors' experiences paralleled reports of others in the existing telehealth literature.

Telehealth offered greater flexibility and increased availability of treatment during a pandemic emergency. Qualitative reports have supported this observation (Knapp et al., 2023). For families seeking specialized care that may live far from a metropolitan area, the cost and time required for travel involved with daily CIMT intervention can impact an entire family.

Telehealth might reduce the financial burden on families in the reduction of time off they have to take to seek specialized care (Pietruszewski et al., 2020). Telehealth could improve access to care for rural populations and patients not near a CIMT site or specialty care (Pietruszewski et al., 2020; Tanner et al., 2020).

Parents also reported that they experienced improved self-efficacy in helping their child and there was a benefit to a coaching model used during telehealth visits. They reported improved ability to facilitate movements in their child's natural environment. There was a benefit to the home environment in this study to improve transfer of motor learning and to engage families within their familiar routines and environments. Overall, the caregivers reported positive experiences with this protocol. One mom reported she, “knew what [type of toys] to shop for” and was able to think more like her child's therapist. Coaching during telehealth appeared to help transfer some clinical reasoning skills to parents. She stated she felt she “got an honorary degree in OT.”

There are clinical considerations for scheduling. Onsite testing ensured the validity of assessments, especially the AHA and QUEST. Further exploration of valid tools that could be completed via telehealth while maintaining sound psychometrics is needed (Trottier et al., 2022). In the clinical experience of these authors parts of the QUEST and the AHA assessments would be easy to execute via telehealth.

In the clinical experience of these authors, ensuring access to toys at home, technology to complete telehealth effectively, and a quiet home environment were essential to care. Identifying barriers and providing solutions would improve telehealth delivery. In some cases, telehealth intervention within the home environment might reduce these barriers and provide for fewer disruptions to a patient and his or her siblings' social and educational needs. Clinically, caregivers have reported difficulty when making the choice to engage in daily, intensive therapy. This is in part due to the changes to the patient's or sibling's schedule or need to forgo peer-based leisure activities given the time commitment required to travel to and complete the therapy regime. Conversely, these authors noted that completing telehealth in a more hectic home environment such as those with multiple children, pets and distractions could negatively impact therapy. One of the patient's caregivers in this report described the patient's brother as jealous of the increased time spent with his sibling on telehealth sessions and leaving the home for a few onsite visits was helpful for the family. This further highlights the need to communicate with families and individualize schedules to their needs. It has also been suggested in the literature to perform a comprehensive needs assessment prior to initiating therapy to identify and problem solve around issues that may arise with scheduling or intensive therapy (Verhaegh et al., 2022).

This telehealth hybrid model worked well for both cases in the Infant CIMT Protocol because it was a one-hour session. With older children who traditionally attend the program for 3 hours this model may not maintain the same therapeutic effect. It may be too challenging to expect parents to manage the child's behavior and to provide hands on assistance to facilitate higher level skills for such a long duration. Caregivers' ability to engage in a 3-hour protocol may also not be practical given other work and household demands.

## Limitations and Considerations

This was a small case series that brought to light considerations for telehealth implementation. Larger studies are needed with sample sizes that reflect the diversity of a clinical population. This case series did not control for normal development and the assessors were not blinded to treatment. In addition, families that engage in telehealth might be more willing to commit to carryover of therapeutic activities at home since they are seeking out treatment, potentially skewing results to a more positive effect.

## Future Directions

Clinically this model may be a conduit to reach families of newly diagnosed infants that live far from specialized care who may not have the means or desire to bring their young child into clinic daily. Telehealth visits may also provide a good way to follow up with discharged patients to help advance or maintain skills achieved during onsite therapy. Practitioners could use telehealth to provide coaching in the patient's natural environment. Development of a decision tree or rubric to determine the factors that might influence choosing an appropriate patient for telehealth or significant barriers to telehealth would also be clinically useful. Significant work and large-scale trials need to be done to study the effectiveness of telehealth visits as a component of CIMT or as a possible treatment delivery method. Although it is far from an established evidence-based intervention, telehealth visits may be a compliment to an existing intervention model that has a robust body of literature supporting its use in pediatric rehabilitation.
